# Ebola research funding: a systematic analysis, 1997–2015

**DOI:** 10.7189/jogh.06.020703

**Published:** 2016-12

**Authors:** Joseph RA Fitchett, Amos Lichtman, Damilola T Soyode, Ariel Low, Jimena Villar de Onis, Michael G Head, Rifat Atun

**Affiliations:** 1 Harvard T.H. Chan School of Public Health, Harvard University, Boston, USA; 2 Global Health Research Institute, University of Southampton, UK

## Abstract

**Background:**

The latest outbreak of Ebola in West Africa overwhelmed the affected countries, with the impact on health extending far beyond Ebola–related deaths that have exceeded 11 000. The need to promptly mobilise resources to control emerging infections is widely recognized. Yet, data on research funding for emerging infections remains inadequately documented.

**Methods:**

We defined research investment as all funding flows for Ebola and/or Marburg virus from 1997 to April 2015 whose primary purpose was to advance knowledge and new technologies to prevent or cure disease. We sourced data directly from funding organizations and estimated the investment in 2015 US dollars (US$).

**Results:**

Funding for Ebola and Marburg virus research in 1997 to 2015 amounted to US$ 1.035 billion, including US$ 435.4 million (42.0%) awarded in 2014 and 2015. Public sources of funding invested US$ 758.8 million (73.1%), philanthropic sources US$ 65.1 million (6.3%), and joint public/private/philanthropic ventures accounted for US$ 213.8 million (20.6%). Prior to the Ebola outbreak in 2014, pre–clinical research dominated research with US$ 443.6 million (73.9%) investment. After the outbreak, however, investment for new product development increased 942.7–fold and that for clinical trials rose 23.5–fold. Investment in new tools to control Ebola and Marburg virus amounted to US$ 399.1 million, with 61.3% awarded for vaccine research, 29.2% for novel therapeutics research such as antivirals and convalescent blood products, and 9.5% for diagnostics research. Research funding and bibliometric output were moderately associated (Spearman’s *ρ* = 0.5232, *P* = 0.0259), however number of Ebola cases in previous outbreaks and research funding (ρ = 0.1706, *P* = 0.4985) and Ebola cases in previous outbreaks and research output (ρ = 0.3020, *P* = 0.0616) were poorly correlated.

**Conclusion:**

Significant public and philanthropic funds have been invested in Ebola and Marburg virus research in 2014 and 2015, following the outbreak in West Africa. Long term, strategic vision and leadership are needed to invest in infections with pandemic potential early, including innovative financing measures and open access investment data to promote the development of new therapies and technologies.

The 2014 Ebola outbreak in West Africa is the largest recorded in history, infecting almost 30 000 individuals by January 2016 and killing over 11 000 people [1].

The challenge with emerging infections is managing uncertainty, as there are many unknown epidemiological and pathophysiological factors. Global surveillance systems are incomplete [2], and health systems responses among interdependent countries vary, putting at risk countries bordering others where the response is weak [3]. Experience responding to the HIV, SARS and Avian Influenza H5N1 epidemics suggests that the cost of inaction, and delayed response to emerging infections, can be significant to human health, the global economy, security and stability [4].

The Ebola outbreak epitomises a largely failed global response, with delayed action by leading international agencies. Weak health systems, a lack of information and all but absent surveillance systems in West Africa among Ebola–affected countries have hampered efforts to control the current outbreak. The lack of a licensed vaccine or effective therapeutic drugs has contributed to the uncontrollable surge in cases and inability to control Ebola transmission beyond traditional infection control practices.

As with infection control measures and strong health systems, research and development (R&D) plays an important role in mitigating risk from emerging infections. Funding for R&D in global health and infectious diseases has risen since 2000 [5]. However, several studies from the US [6], United Kingdom (UK) [7], Spain [8], Australia [9] and Norway [10] suggest low levels of R&D funding and a lack of reliable data for neglected diseases and low–income settings [11]. Research also indicates a paucity of funding for other conditions for which there is no sizeable market, for example women during pregnancy and neonates [12,13].

More recently, studies have presented systematic analysis of public and philanthropic financing of infectious disease research in the UK to show a predominant focus on preclinical and laboratory research across a wide range of infections and crosscutting disciplines such as diagnostics, therapeutics, and vaccines [7]. Research funding, however, was not well aligned to disease burden and followed colonial ties rather than need [14]. To our knowledge, there have been no studies quantifying funding for filovirus research.

We present the first systematic analysis of global funding for research and emergency response for Ebola and Marburg virus infections. The primary purpose of the study is to quantify global research funding for filovirus research prior to, and following, the largest recorded outbreak of Ebola virus.

## METHODS

Measures of research investment were sourced directly from funding organizations, data on disease burden from the Global Burden of Disease Study 2012, and bibliometric impact from the Elsevier Scopus database. The study forms part of a wider project entitled RESIN: Research Investments in Global Health. A full list of keywords, definitions, categories, sources of funding and data sets are available online (http://www.researchinvestments.org/ebola) and in Appendix S1 of the **Online Supplementary Document(Online Supplementary Document)**.

We included studies that focused on Ebola infection in humans, or animal studies with a clear zoonotic component. For completeness, we also screened and systematically analyzed research investments for Marburg virus and Cuevavirus, two other related filoviruses, as funding for these viruses are often joint. No studies on filovirus Cuevavirus were identified, and it was therefore excluded from the scope of this analysis.

### Data sources and collection

We sourced information from websites, funding organization databases, and the published literature. Data from the UK, European Union (EU) and the US were included for the period of 1997 to 16 April 2015, which represented a 12–month period following the announcement of the outbreak by the WHO. Variables collected included study title, abstract, website, grant type, funding awarded, name and gender of the principal investigator, host institution, year of award and projected duration of project.

In the UK, research funding organisations included the UK Medical Research Council (MRC), the Wellcome Trust, the UK Department for International Development (DFID); in the EU organisations included the European Commission, the European Centre for Disease Prevention and Control (ECDC); and in the US organisations included the National Institutes of Health (NIH), Congressionally Directed Medical Research Programmes (CDMRP), Bill and Melinda Gates Foundation, Paul G. Allen Foundation, Burroughs Wellcome Fund, Doris Duke Charitable Foundation, Howard Hughes Medical Institute, Donaghue Foundation, Ellison Medical Foundation, Arcus Foundation, and the Roy Carver Charitable Trust. The organisations selected represented the leading infectious disease and public health research funders in the respective regions. Variables collected included financial disbursements, project title, website, donor organization, recipient organization, recipient country, and year of award.

### Data management

All grant funding amounts were reported in 2015 US dollars (US$). All awards were adjusted for inflation and converted to US dollars, using the mean exchange rate in the year award (http://www.oanda.com). Grants were not modified according to levels of overheads applied to the award. For multi–center studies, the distribution of funding was accounted for, where openly available. Unfunded studies, or studies without a clear funding amount, were excluded. Private sector sources of funding were excluded, as data was not openly available. Channels without a robust data source were excluded from the final analysis.

Data were collected over a period of 7 months, from October 2014 to April 2015. The study title, abstract, and website were used to filter and categorise research studies. All research studies were reviewed by two or more co–authors. Each research award was allocated to one of five R&D categories along with the research pipeline: preclinical research, phase 1, 2, or 3 clinical trials, product development, public health research, and cross–disciplinary research. Public health research included surveillance, epidemiology, modeling, bioinformatics, and operational research. Cross–disciplinary studies were large–scale projects, with significant funds to facilitate two or more subprojects to work in parallel. Duration of research studies was also estimated. Top 3 donors were ranked for each recipient organization.

### Statistical analysis

Microsoft Excel 2011 (Microsoft, Seattle WA, USA) was used to categorise the research studies and generation of tables. Statistical analysis and generation of figures and graphs were generated using Stata (version 11.2) (STATA Corp LLP, College Station, Texas, USA). Simple regression analyses were reported using Spearman’s rank correlation coefficient (*ρ)*, to assess the degree of correlation between research investment, disease burden, and bibliometric impact. We used fold differences to compare total investment, number of studies, mean and median grant size.

### Role of the funding source

There was no funding source for this study. The corresponding author had full access to all the data in the study and had final responsibility for the decision to submit for publication.

## RESULTS

**Figure 1:**
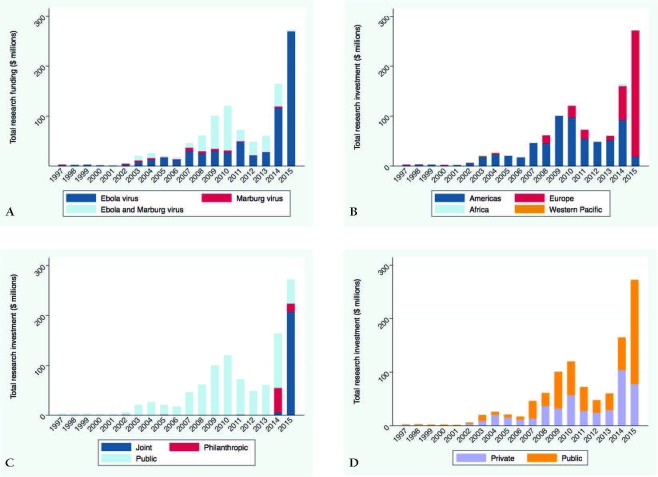
Total and proportionate investment in research funding by filovirus (A), by geographical location of lead research institution (B), by source of funding (C), and by recipient of funding (D), in 2015 US$, 1997–2015.

**Figure 1** shows the total research investment in Ebola and Marburg virus from 1997 to 2015 in US$, disaggregated by virus, location of award, source of funding, and recipient of funding, respectively.

The total research funding awarded over the 18–year time period was US$ 1.035 billion, including US$ 435.4 million (42.0%) disbursed in 2014 and 2015 during the West Africa Ebola outbreak. Ebola virus received US$ 652.4 million (63.0%), Marburg virus received US$ 34.9 million (3.4%) and cross–cutting filovirus research received US$ 348.3 million (33.6%).

Relative contributions by Ebola and Marburg virus changed substantially over time, with significant increases in 2003 (from US$ 5.2 million in 2002 to US$ 19.4 in 2003), in 2007 (from US$ 16.5 million in 2006 to US$ 45.7 million in 2007) and in 2014 (from US$ 59.6 million in 2013 to US$ 164.0 million in 2014) respectively (**Figure 1**).

There is clear dominance in research funding awarded by the US, with large rises in 2014 and the 1st quarter of 2015. Large proportions of global research funding are invested in institutions in the European Union, such as the UK with a total of US$ 152.5 million, Germany with a total of US$ 66.0 million, and France with a total of US$ 49.4 million over the 15–month period January 2014–April 2015. Institutions in the US were awarded a total of US$ 110.1 million during the same time period, predominantly in 2014 (83.2%) (**Table 1**).

**Table 1:** Investments by public, private and philanthropic funders for Ebola research

	Funding awarded, US$	%	Type of organization	Funding received, US$	%	Ratio investment:award
**Public funding:**	756 792 968	73.1	**Public institution:**	346 738 377	33.5	1:0.46
NIH/NIAID	651 044 589	62.9	University	291 139 730	28.1	
European Commission	93 822 622	9.1	Research Institute	212 219 525	20.5	
German government	4 996 424	0.5	Public Health Institute	63 925 988	6.2	
CDC PHPR	4 634 854	0.4	**Non–profit organization:**	455 291 509	44.0	1:7
CDMRP	2 232 070	0.2	University	287 955 319	27.8	
**Philanthropic funding:**	65 064 309	6.3	Research Institute	80 249 373	7.7	
Gates Foundation	52 713 304	5.1	Public Health Institute	60 059 515	5.8	
Wellcome Trust	11 085 919	1.1	NGO	20 765 944	2.0	
Burroughs Wellcome Fund	601 000	0.1	Hospital	6 261 358	0.6	
Ellison Medical Foundation	263 200	0.0	**For–profit organization:**	233 591 124	22.6	1:2
Paul G. Allen Foundation	170 416	0.0	Biopharmaceuticals	229 370 939	22.1	
Arcus Foundation	121 832	0.0	Research Institute	3 491 497	0.3	
Roy Carver Charitable Trust	108 638	0.0	Technology	728 688	0.1	
**Joint funding:**	213 763 733	20.6				
EFPIA	114 980 222	11.1				
European Commission	90 797 873	8.8				
Wellcome Trust/DFID/MRC	8 048 047	0.8				
**Total Ebola research funding**	**1 035 621 010**	**100**				

CDMRP – Congressionally Directed Medical Research Programme, CDC PHPR – CDC Office of Public Health Preparedness and Response, NIH – National Institutes of Health, NIAID – National Institute of Allergy and Infectious Diseases, EFPIA – European Federation of Pharmaceutical Industries and Associations, DFID – UK Department for International Development

Public sources of funding accounted for the majority of total investment in Ebola and Marburg research with US$ 758.8 million (73.1%). Philanthropic sources awarded US$ 65.1 million (6.3%), and joint public–philanthropic or public–private funding accounting for US$ 213.8 million (20.6%), including a further US$ 90.8 million by the European Commission (**Table 1**).

Publicly funded institutions received a total of US$ 346.7 million (33.5%) research funding. Private, for profit institutions received a total of US$ 233.6 million (22.6%) and philanthropic, non–profit institutions received a total of US$ 455.3 million (44.0%). In other words, for every US$ 1 invested by a public funding source, a public institution received US$ 0.46. For every US$ 1 invested by a private funding source, a for–profit organization received US$ 2.03, and for every US$ 1 invested by a philanthropic funding source, a non–profit organization received US$ 7.00. Universities received the largest investment in Ebola and Marburg virus disbursements with US$ 579.1 million (55.9%) followed by Research Institutions with US$ 296.0 million (28.6%) and biopharmaceutical companies with US$ 229.3 million (22.1%).

**Figure 2:**
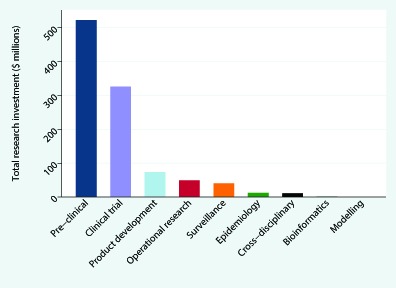
Total investment in Ebola and Marburg research by R&D pipeline, in 2015 US$, 1997–2015.

**Figure 2** shows the research investment according to type of research along the R&D pipeline between 1997–2015. Prior to the outbreak, pre–clinical research dominated research with US$ 443.6 million (73.9%) investment. Per annum investment prior to the Ebola outbreak in West Africa, annual research funding was US$ 35.3 million over a period of 17 years. Following the outbreak, annual research funding increased 9.5–fold to US$ 337.1 million.

**Figure 3:**
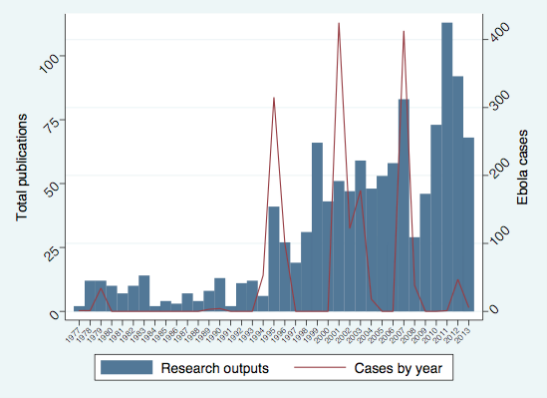
Research bibliometrics output over time with total Ebola cases per annum between 1977–2013.

In 2014 and 2015, a 942.7–fold increase in product development and 23.5–fold increase in clinical trials was observed (**Table 2**). Analyzing data from 1997–2013, there was a moderate association between research funding and research output (ρ = 0.5232, *P* = 0.0259), however Ebola cases in previous outbreaks and research funding (ρ = 0.1706, *P* = 0.4985) and Ebola cases in previous outbreaks and research output (ρ = 0.3020, *P* = 0.0616) were poorly correlated (**Figure 3**). Investment in innovative technologies for Ebola and Marburg virus amounted to US$ 399.1 million, with 61.3% awarded for vaccine research, 29.2% for novel therapeutics research such as antivirals and convalescent blood products, and 9.5% for diagnostics research (**Table 3**).

**Table 2:** Investment in R&D pipeline by focus of Ebola research

	1997–2013			2014–2015			
	**US$**	**%**	**Per annum**	**US$**	**%**	**Per annum**	**Fold difference**
**Preclinical:**	443 570 456	73.9	26 092 380	78 755 577	18.1	60 972 060	2.3
Host–pathogen	255 676 031	42.6	15 039 767	21 317 338	4.9	16 503 746	1.1
Non–human primates	204 216 041	34.0	12 012 708	16 560 952	3.8	12 821 382	1.1
**Clinical trials:**	116 942 041	19.5	6 878 944	208 852 606	48.0	161 692 340	23.5
Phase 1	116 942 041	19.5	6 878 944	153 285 276	35.2	118 672 472	17.3
Phase 2–3	–	–	–	55 567 330	12.8	43 019 868	–
**Product development:**	1 015 328	0.2	59 725	72 726 099	16.7	56 304 077	942.7
**Cross–disciplinary:**	11 057 065	1.8	650 416	–	0.0	–	–
**Public health research:**	27 581 023	4.6	1 622 413	75 120 815	17.3	58 158 050	35.8
Surveillance	19 840 535	3.3	1 167 090	20 260 954	4.7	15 685 900	13.4
Epidemiology	6 329 902	1.1	372 347	5 795 974	1.3	4 487 206	12.1
Modeling	501 655	0.1	29 509	433 870	0.1	335 899	11.4
Bioinformatics	908 931	0.2	53 467	224 085	0.1	173 485	3.2
Operational research	–	–	–	48 405 932	11.1	37 475 560	–
**Subtotal**	600 165 913	100	35 303 877	435 455 097	100	337 126 527	9.5
**Total research funding**	**1 035 621 010**						

**Table 3:** Investment in innovation in Ebola research by type of institution

	Vaccines		Diagnostics		Therapeutics			
	**US$**	**%**	**US$**	**%**	**US$**	**%**	**Total**	**%**
Research Institute	108 615 512	44.4	7 832 065	20.6	21 464 466	18.5	137 912 043	34.6
Biopharmaceuticals	93 932 058	38.4	9 861 635	25.9	27 752 433	23.9	131 546 126	33.0
University	30 429 464	12.4	20 046 125	52.6	60 181 420	51.7	110 657 009	27.7
NGO	11 667 539	4.8	–	–	1 376 869	1.2	13 044 408	3.3
Hospital	–	–	–	–	5 466 616	4.7	5 466 616	1.4
Public Health Institute	–	–	369 008	1.0	94 611	0.1	463 619	0.1
**Subtotal**	244 644 573	61.3	38 108 833	9.5	116 336 415	29.2	399 089 821	38.5
**Total research funding**	**1 035 621 010**							

## DISCUSSION

### Implications of mapping research funding

Emerging infections have the potential to disrupt the global economy and global health, mobilising significant resources over short periods of time. In an effort to learn from the latest Ebola outbreak in West Africa and prevent the next epidemic, data on current investments, coupled with data on disease burden and efficacy of interventions, may provide an enabling environment to strategically allocate scarce resources [15].

We demonstrate significant public and philanthropic funds have been invested in Ebola and Marburg virus research in 2014 and 2015.

Evaluating the impact of R&D is not only good science, but also a social imperative. The responsibility to invest appropriately lies both with researchers, and with funding organisations. Policymakers must work closely with the scientific and funding communities to facilitate channels that provide both the flexibility and the strategic resources to alleviate the burden of emerging infections. Evidence–informed investment is key to allocating resources wisely and fairly. Our analyses provide a first step in aggregating and describing the trends with Ebola and Marburg viruses, in an effort to develop more sensitive methods to evaluate the public health impact of supported research.

What the Ebola crisis in West Africa has demonstrated is the immense repercussions of an outbreak on the stability and social cohesion of a society. The spread of the outbreak is linked to the under–developed and under–resourced health systems preexisting within these countries, but also the imbalance between investments in the diseases that affect individuals in wealthier countries over those living in low–income settings [16].

### Proposals for research strategy

New approaches for research during a new epidemic or pandemic are critical, and useful examples include the 2009 NIHR “Rapid Response” research funding for influenza [17], and the 2011 NIHR “Sleeping grants” where protected funding will be activated in the event of a new pandemic [18].

A portfolio of research along the R&D value chain (extending from preclinical and laboratory science, phase 1 and phase 2 clinical trials, large–scale phase 3 and phase for clinical trials, translational studies and evaluations) is required to address the greatest challenges in global health. Investing in the progression and links between these different types of research is essential in order to build on early stage research findings, and translate breakthroughs in the laboratory into reality in health systems where translation and uptake of innovations remains a challenge [19,20]. Operations research is critical to understand how innovations can be effectively scaled up [21,22].

Encouragingly, the scope and volume of potential Ebola diagnostics, therapies and vaccines is broadening (**Box 1**).

Box 1 **Summary of current research for Ebola diagnostics, therapies and vaccines.**

Research is currently under way to investigate the therapeutic potential of favipiravir (Toyama Chemical, Japan) and convalescent whole blood and plasma treatment [23]. Clinical trials in Liberia of brincidofovir (Chimerix; NC, USA) with Médecins Sans Frontières and the Wellcome Trust were discontinued due to paucity of numbers recruited. Should these front–runners fail to show promise, 10–15 pre–qualified products may become the next candidates. Zmapp (Mapp Biopharmaceuticals; CA, USA) was not selected as part of the early research candidates due to availability issues.

Efficacy trials for an Ebola vaccine have begun in February 2015, 14 months after the estimated start of the outbreak, using a randomized ring vaccination design adopted with the smallpox eradication campaign [24]. In clarifying their position, Gavi, the Vaccine Alliance, has approved US$ 300 million spending on vaccine procurement and US$ 90 million on strengthening immunisation systems [25]. The overarching aim is to enable vaccine development for emerging infections by recognizing the lack of market potential, building laboratory and outbreak investigation capabilities in country, create repositories of potential agents and catalog immunological properties, and develop vaccine vectors. Two candidates exist: GlaxoSmithKline's ChAd3–ZEBOV and Merck's rVSV–Ebov. Further preclinical vaccine candidates include Johnson & Johnson's recombinant vector regimen, a recombinant nanoparticle vaccine by Novavax, a recombinant influenza vaccine developed by the Russian Ministry of Health, a Venezuela equine encephalitis replicon Ebola vaccine developed by the US Army Medical Research Institute for Infectious Diseases, and possibly a further vaccine developed by the Chinese Army. The imperative to invest in emerging infections or diseases with high mortality rates lies in the fact that suboptimal evidence may be generated during an outbreak, due to the urge to develop therapies without randomized controlled trials. Randomization is essential in Ebola drug trials, as it is with cancer trials [26]. An assumption that a therapy will be effective is inappropriate for generating evidence, and harm may be the price of introducing this bias. Strategic health investment can go a long way to ensuring sufficient innovation in science is being fostered, preventing a reliance on insufficient and potentially wasteful products toward the end of the R&D pipeline.

R&D should also extend beyond vaccines, diagnostics, and antivirals, however. Research into novel, digital surveillance systems is also warranted. Surveillance systems are essential to detect outbreaks of emerging infections, and to mitigate their health, security and economic effects, in all countries [27,28]. The International Health Regulations (IHR) confers responsibilities to WHO member states to develop surveillance systems to detect and respond to public health emergencies, although many are not ready [29]. IHR provides a mandate for global surveillance, a safety net to detect disease outbreaks if they are not detected and/or reported by countries. Responsive health systems and global collaboration is critically important if these infections are to be rapidly contained [30,31]. In 2002, the Global Public Health Intelligence Network, developed by the Public Health Agency of Canada, openly alerted the global health community to SARS 2 months prior to the WHO. In 2014, HealthMap sounded the alarm to an outbreak of unknown etiology in Guinea 9 days before the WHO. Recent work has further highlighted the density of Google Trends searches for Ebola in West Africa, despite inadequate Internet coverage in the region [32]. These factors together highlight the need for research into digital systems for global health security.

### Limitations and scope

The primary limitation to this work is the difference in quality of research funding, compared with emergency response funding. Although public and philanthropic research funding is, on the whole, well documented and often openly accessible online, there are clear quality concerns with emergency response funding. For instance, it is difficult to verify disbursements and pledges for emergency response, and this is a notable problem with the latest Ebola outbreak. As published in the Lancet Global Health – the tools “for tracking resources in a crisis are not fit for purpose” [33]. Despite the challenges with emergency response funding, it is important to document the openly published information, systematically analyze the data and start a discussion on next steps in tracking resources during a crisis. We need a robust financial platform to monitor and evaluate investments – with the capacity to cross–talk with research funding commitments. With the re–emergence of Zika virus in the Americas, this needs to occur without delay.

There are also limitations to analyzing global expenditure data search, elaborated in greater detail in a recent article by Young and colleagues [34]. The first is the challenge of comparing data collected from different countries. The Frascati manual attempts to provide some guidance on the data collection and currency adjustments for R&D [35]. Despite some clear guidance, two recent studies highlight the ongoing difficulties with methodology and subsequent impact on interpretation, particularly due to discrepancies in data conversion [36–38]. The second is the lack of indices to convert health R&D expenditure to a single, commonly used currency [38]. One of the main reasons this is important is the comparative difference in the costs of input for R&D in different settings [39]. There are two possible ways of converting to a common currency: current exchange rates, and GDP purchasing power parities (PPPs) [40]. The Frascati manual recommends GDP PPPs, and current exchange rates may underestimate the contributions of countries such India and China, by overstating the costs of R&D. The third is the need to adjust investment over time, in order to account for the potential role of inflation. There is currently no index specific for health R&D and the Frascati manual advocates for using the GDP price index, with the limitation that is not specific for R&D. The special R&D deflator is an index adopted by the US National Institutes of Health and may be adopted for high–income settings [41]. Using this index for settings with high inflation will overestimate respective contributions, and vice versa. The fourth challenge relates to the need first to deflate data in national currency, then to covert the adjusted data to a common currency using a selected base year [42]. Altering data using another mechanism may skew the data inappropriately and alter the comparability between settings. Fifth, our study is likely to underestimate research funding, partly due to the lack of openly available data from the pharmaceutical industry. Sixth challenge relates to difficulties in ascertaining the right proportion of a grant allocated to a specific disease, when there are multiple research sites and conditions researched, which is not uncommon for co–infection studies.

### Strategic coordination of research funding

A Global Observatory on health R&D has been proposed by member states of the sixty–sixth World Health Assembly in 2013 to “provide a mechanism to monitor and analyse relevant existing information on health R&D, including resource flows, product pipelines, and research outputs, with a view to contributing to the identification of gaps and opportunities for health R&D and to inform priority–setting for new R&D investments based on the public health needs of the world’s poorest countries” [43,44].

A clear, open source repository for R&D investment data could address the problem by showing the funding landscape of both clinical and non–clinical studies in real time [45]. Gaps in the data highlight the need for a global health R&D observatory. Innovations in reporting will help improve priority setting to address burden of disease in low– and middle–income settings. Interpreting disease burden data in the context of R&D funding data are an essential step in the equitable allocation of health investments. Whether the observatory should be run by the WHO or by another institution or consortium, however, remains for debate.

## CONCLUSIONS

Over 20 years after a landmark publication by the Institute of Medicine, emerging and re–emerging infectious diseases continue pose a serious threat globally to human and animal health, security and economy [46]. Health and financial resources need to be allocated and available swiftly during a pandemic or emerging infectious disease outbreak to ensure appropriate control. The global health community needs to develop an evidence–base for health research and policy, in particular around priority setting for health R&D, decision making for funding, evaluating innovation and research pipelines, assessing research outputs and networks, and forecasting future needs and corresponding investment. Strategic funding is required for inter–pandemic research, pre–pandemic research and established pandemic research.
