# Supplementary material for: Ebola research funding: a systematic analysis, 1997–2015
Source: J Glob Health. 2016 Nov 9;6(2):020703. doi: 10.7189/jogh.06.020703 (PMC5112007; doi:10.7189/jogh.06.020703)
Supplement: Online Supplementary Document [file jogh-06-020703-s001.pdf]

Online Supplementary Document

Fitchett et al. Ebola research funding: a systematic analysis, 1997–2015

J Glob Health 2016;6:020703

Table S1. EU Ebola research investments, 1997-2013

Year	Project title	R&D pipeline	Research focus	Filovirus	Funding organisation
1997	Studies on Marburg virus reproduction in different cell systems	Pre-clinical	Host-pathogen	Marburg virus	European Commission
1998	Adaptation and field evaluation of an RT-PCR technique to detect the RNA genomes of haemorrhagic fever viruses	Pre-clinical	Diagnostics	Ebola and Marburg virus	European Commission
1998	Surveillance and research project on viral hemorrhagic fevers in West Africa	Translational	Surveillance	Ebola virus	European Commission
2000	Interaction between components of the cytoskeleton and Marburg and Ebola virus nucleocapsids in infected cells	Pre-clinical	Host-pathogen	Ebola and Marburg virus	European Commission
2002	Biophysical and biochemical studies of the L polymerase of Ebola virus	Pre-clinical	Host-pathogen	Ebola virus	Wellcome Trust
2002	Molecular interactions in filovirus assembly and budding	Pre-clinical	Host-pathogen	Ebola virus	European Commission
2004	Development and commercial production of standardized PCR-assays for detection of hemorrhagic fever viruses and variola virus	Product development	Diagnostics	Ebola and Marburg virus	European Commission
2010	From bats to humans: the social, ecological and biological dynamics of pathogen spillover	Translational	Surveillance	Ebola and Marburg virus	Medical Research Council
2011	ANTIGONE: Anticipating the Global Onset of Novel Epidemics	Translational	Surveillance	Ebola virus	European Commission
2013	Identification of Host Determinants for Virus Entry using a Haploid Genetic Approach	Pre-clinical	Host-pathogen	Ebola virus	European Commission

Table S2. EU Ebola research investments, 2014-15

Year	Project title	R&D pipeline	Research focus	Filovirus	Funding organisation	Host institution	Country	Gender of principal investigator	Funding ($)
2014	EVIDENT: correlates of protection, determinants of outcome and clinical management	Pre-clinical	Host-pathogen	Ebola virus	European Commission	Bernhard Nocht Institute for Tropical Medicine, Hamburg	Germany	Male	$2,378,679
2014	Analysing and inhibiting Ebola virus entry into host cells	Pre-clinical	Host-pathogen	Ebola virus	Federal Ministry of Education and Research	German Primate Centre, Göttingen	Germany	Male	$777,423
2014	Using chimeric mouse models to investigate Ebola virus immunity and pathogenesis	Pre-clinical	Host-pathogen	Ebola virus	Federal Ministry of Education and Research	Bernhard Nocht Institute for Tropical Medicine, Hamburg	Germany	Male	$777,423
2014	IF-EBOla	Pre-clinical	Therapeutics	Ebola virus	European Commission	French Institute of Research and Development (IRD), Marseille	France	Male	$2,694,305
2014	Rapid down-selection of experimental therapies for Ebola Virus disease	Pre-clinical	Therapeutics	Ebola virus	Wellcome Trust	Public Health England, London	United Kingdom	Male	$335,136
2014	Developing MVA vector vaccines to prevent Ebola virus infections	Pre-clinical	Vaccines	Ebola virus	Federal Ministry of Education and Research	Leopold Maximilian University, Munich	Germany	Male	$777,423
2014	Developing and validating pan-Ebola vaccination strategies	Pre-clinical	Vaccines	Ebola virus	Federal Ministry of Education and Research	Paul-Ehrlich Institute, Langen	Germany	Female	$777,423
2014	Development of a social marketing strategy to promote Ebola treatment-seeking behaviour in Sierra Leone	Operational research	Health systems	Ebola virus	Wellcome Trust / DFID	Umea University	Sweden	Male	$264,757
2014	Investigating a possible secondary reservoir in animals in West Africa	Operational research	Surveillance	Ebola virus	Federal Ministry of Education and Research	Robert Koch Institute, Berlin	Germany	Male	$143,457
2014	Developing adaptive, interactive software to assess absolute risk of Ebola imports in global air traffic network hubs	Operational research	Surveillance	Ebola virus	Federal Ministry of Education and Research	Robert Koch Institute, Berlin	Germany	Male	$137,995
2014	Developing, implementing and evaluating a follow-up tool for staff in Ebola treatment centres and returning travellers from Ebola epidemic regions	Operational research	Surveillance	Ebola virus	Federal Ministry of Education and Research	Robert Koch Institute, Berlin	Germany	Male	$143,457
2014	Ebola surveillance with mobile real-time data transmission in Nigeria	Operational research	Surveillance	Ebola virus	Federal Ministry of Education and Research	Helmholtz Centre for Infection Research, Braunschweig	Germany	Male	$908,571

2014	Reducing Ebola virus transmission: Improving contact identification and tracing in Sierra Leone	Operational research	Surveillance	Ebola virus	German Federal Enterprise for International Cooperation (GIZ)	London School of Hygiene & Tropical Medicine	United Kingdom	Male	$553,252
2014	Predicting the geographical spread of Ebola virus disease in West Africa	Operational research	Surveillance	Ebola virus	Wellcome Trust / DFID	University of Oxford	United Kingdom	Male	$159,489
2014	Modelling Ebola in West Africa	Operational research	Modelling	Ebola virus	Wellcome Trust / DFID	London School of Hygiene & Tropical Medicine	United Kingdom	Male	$433,870
2014	REACTION	Clinical trial: Phase 2-3	Therapeutics	Ebola virus	European Commission	French Institute of Health and Medical Research (INSERM), Paris	France	Male	$3,479,894
2014	Ebola_Tx	Clinical trial: Phase 2-3	Therapeutics	Ebola virus	European Commission	Institute of Tropical Medicine, Antwerp	Belgium	Male	$3,910,331
2014	EbolaVac	Clinical trial: Phase 1	Vaccines	Ebola virus	European Commission	GlaxoSmithKline Biologicals	Belgium	data not available	$20,487,755
2014	Coordinated Clinical Trials of VSV-Ebola Virus Vaccine	Clinical trial: Phase 1	Vaccines	Ebola virus	Wellcome Trust	University of Oxford	United Kingdom	Female	$5,231,476
2014	Accelerated Clinical Evaluation of a Monovalent Vectored Ebola vaccine	Clinical trial: Phase 1	Vaccines	Ebola virus	Wellcome Trust / DFID / MRC	University of Oxford	United Kingdom	Male	$4,725,416
2015	Mofina project	Product development	Diagnostics	Ebola and Marburg virus	European Commission	Altona Diagnostics GmbH, Hamburg	Germany	data not available	$1,140,677
2015	FILODIAG	Product development	Diagnostics	Ebola virus	European Commission	GNA Biosolutions GmbH, Planegg	Germany	Male	$2,623,557
2015	EbolaMoDRAD	Product development	Diagnostics	Ebola virus	European Commission	Public Health Agency of Sweden, Stockholm	Sweden	Male	$4,904,910
2015	Ebolacheck	Product development	Diagnostics	Ebola virus	Wellcome Trust / DFID	University of Westminster, London	United Kingdom	Male	$1,046,316
2015	Pointof-care diagnostic testing for Ebola virus disease in Ebola treatment centers	Product development	Diagnostics	Ebola virus	Wellcome Trust / DFID	Institut Pasteur, Dakar	Senegal	Male	$769,350
2015	EBOMAN	Product development	Vaccines	Ebola virus	European Commission	Vibalogics GmbH	Germany	data not available	$1,140,677
2015	EBOMAN	Product development	Vaccines	Ebola virus	European Federation of Pharmaceutical Industries and Associations	Vibalogics GmbH	Germany	data not available	$54,296,216

2015	Developing fluorescing recombinant Ebola viruses (Guinea strain) to enable rapid testing of emerging mutations in the virus genome and their pathogenic implications	Pre-clinical	Host-pathogen	Ebola virus	Federal Ministry of Education and Research	Philipps University, Marburg	Germany	Male	data not available
2015	Drug discovery partnership to produce an antibody-based medicine to fight Ebola infection	Pre-clinical	Therapeutics	Ebola virus	Wellcome Trust	Kymab, Cambridge	United Kingdom	Male	$538,545
2015	Systems vaccinology: Hereditary predictors of Ebola virus induced adaptive immunity	Pre-clinical	Vaccines	Ebola virus	Federal Ministry of Education and Research	University Medical Centre, Hamburg	Germany	Female	data not available
2015	EBODAC	Operational research	Health systems	Ebola virus	European Commission	London School of Hygiene & Tropical Medicine	United Kingdom	data not available	$23,155,739
2015	EBODAC	Operational research	Health systems	Ebola virus	European Federation of Pharmaceutical Industries and Associations	London School of Hygiene & Tropical Medicine	United Kingdom	data not available	$6,159,655
2015	Ebola Response Anthropology Platform	Operational research	Health systems	Ebola virus	Wellcome Trust / DFID	London School of Hygiene & Tropical Medicine	United Kingdom	Female	$307,740
2015	Participatory behavioural change to reinforce infection prevention and control for Ebola virus disease in Sierra Leone	Operational research	Health systems	Ebola virus	Wellcome Trust / DFID	International Rescue Committee	United Kingdom	Female	$278,700
2015	Investigating the filovirus transmission chain in an industrialised West African country	Operational research	Surveillance	Ebola virus	Federal Ministry of Education and Research	University of Bonn	Germany	Male	data not available
2015	EBOVAC2	Clinical trial: Phase 2-3	Vaccines	Ebola virus	European Commission	French Institute of Health and Medical Research (INSERM), Paris	France	Male	$26,007,431
2015	EBOVAC2	Clinical trial: Phase 2-3	Vaccines	Ebola virus	European Federation of Pharmaceutical Industries and Associations	French Institute of Health and Medical Research (INSERM), Paris	France	Male	$17,224,220
2015	Emergency Evaluation of Treatments for Ebola Virus Disease	Clinical trial: Phase 2	Therapeutics	Ebola virus	Wellcome Trust	University of Oxford	United Kingdom	Male	$4,945,454
2015	VSV-EBOVAC	Clinical trial: Phase 1	Vaccines	Ebola virus	European Commission	Sclavo Vaccines Association	Italy	Female	$4,448,640
2015	EBOVAC1	Clinical trial: Phase 1	Vaccines	Ebola virus	European Commission	London School of Hygiene & Tropical Medicine	United Kingdom	data not available	$66,501,457
2015	EBOVAC1	Clinical trial: Phase 1	Vaccines	Ebola virus	European Federation of Pharmaceutical Industries and Associations	London School of Hygiene & Tropical Medicine	United Kingdom	data not available	$37,300,131

2015	Conducting a phase I VSV-Ebola vaccination trial	Clinical trial: Phase 1	Vaccines	Ebola virus	Federal Ministry of Education and Research	University Hospital of Tübingen	Germany	Male	data not available
